# Supplementary material for: Determinants of Burnout among Teachers: A Systematic Review of Longitudinal Studies
Source: Int J Environ Res Public Health. 2022 May 9;19(9):5776. doi: 10.3390/ijerph19095776 (PMC9104901; doi:10.3390/ijerph19095776)
Supplement: Supplementary file 1 [file ijerph-19-05776-s001.zip › ijerph-1689120-supplementary/ijerph-1689120-Supplementary-done.pdf]

International Journal of
Environmental Research and Public Health

Supplementary Table S1. Standardized regression coefficients and standard errors for the burnout determinants included in the category *support*

article	support	wave	school	beta	se
Beausert_2016	collegue	1	primary	0.003	0.035
Beausert_2016	supervisor	1	primary	0.023	0.027
Beausert_2016	community	1	primary	0.066	0.028
Beausert_2016	collegue	2	primary	-0.051	0.023
Beausert_2016	supervisor	2	primary	0.023	0.021
Beausert_2016	community	2	primary	0.076	0.023
Beausert_2016	collegue	3	primary	0.034	0.023
Beausert_2016	supervisor	3	primary	-0.029	0.022
Beausert_2016	community	3	primary	0.059	0.025
Beausert_2016	collegue	1	secondary	-0.011	0.056
Beausert_2016	supervisor	1	secondary	0.018	0.044
Beausert_2016	community	1	secondary	0.06	0.047
Beausert_2016	collegue	2	secondary	-0.133	0.042
Beausert_2016	supervisor	2	secondary	-0.021	0.036
Beausert_2016	community	2	secondary	0.12	0.043
Beausert_2016	collegue	3	secondary	-0.027	0.046
Beausert_2016	supervisor	3	secondary	-0.009	0.042
Beausert_2016	community	3	secondary	-0.039	0.051
Feuerhahn_2013	emotional	NA	NA	-0.21	0.14
Salanova_2005	social facilitator	NA	NA	0.03	0.05

Supplementary Table S2. Standardized regression coefficients and standard errors for the burnout determinants included in the category *conflict*

article	type	beta	se
Feuerhahn_2013	parents criticism	0.24	0.16
Feuerhahn_2013	conflict with collegues	0.01	0.13
Salanova_2005	parents_students	-0.01	0.04

Supplementary Table S3. Standardized regression coefficients and standard errors for the burnout determinants included in the category *individual characteristics*

article	type	beta	se
Feuerhahn_2013	emotional_dissonance	0.02	0.17
Feuerhahn_2013	teacher_self_efficacy	0.68	0.24
Malinen_2016	teacher_self_efficacy	-0.36	-0.109
Malinen_2016	job_satisfaction	-0.28	-0.085
Salanova_2005	exhaustion_t1	0.81	0.04
Goddard_2006	neuroticism	0.19	0.061

Supplementary Table S4. Standardized regression coefficients and standard errors for the burnout determinants included in the category *organizational context*

article	type	beta	se
Feuerhahn_2013	time_pressure	-0.03	0.13
Feuerhahn_2013	classroom_disruption	0.38	0.18
Gonzales-Morales_2012	perceived_collective_exhaustion	0.33	0.15
Gonzales-Morales_2012	workload_stressors	0.09	0.07
Salanova_2005	technical_obstacles	0.01	0.04
Salanova_2005	effective_class_management	-0.07	0.07
Goddard_2006	work_climate	0.29	0.09
